# QR Codes in medical education: A mixed-method for evaluation of effectiveness and learner perception

**DOI:** 10.6026/973206300214250

**Published:** 2025-11-15

**Authors:** Puja Singh, Maneesh Jain, Jitendra Dangi

**Affiliations:** 1 Department of Pathology, Bundelkhand Medical College, Sagar, Madhya Pradesh, India; 2 Department of Medicine, Bundelkhand Medical College, Sagar, Madhya Pradesh, India; 3 Department of Surgery, Bundelkhand Medical College, Sagar, Madhya Pradesh, India

**Keywords:** QR code, teaching and learning tool, medical education

## Abstract

Leveraging QR codes in medical education enhances student motivation, autonomy and engagement. Therefore, it is of interest to evaluate the use of QR codes as educational tools among 106 Phase 3 MBBS students in India, utilizing online resources such as lecture slides and quizzes. Data collected through surveys and focus group discussions revealed that 98.11% of participants were familiar with QR codes. Data indicated improved engagement and convenience in accessing educational content, although opinions varied on their comfort and effectiveness compared to traditional methods. While QR codes enrich learning experiences, challenges like technology access and internet connectivity must be addressed.

## Background:

Technology integration in medical education has transformed traditional learning paradigms, making innovative tools essential for enhancing student engagement and outcomes. Quick Response (QR) codes, developed initially for retail and inventory management, have become influential in educational settings due to their ability to link physical and digital environments seamlessly [1]. Particularly in medical training, where access to diverse educational resources is critical, QR codes enable instant access to multimedia content and interactive learning experiences [2]. This study seeks to evaluate the effectiveness and perceptions of QR codes as teaching-learning tools among Phase 3 MBBS students at a medical college in Central India. Existing literature highlights the positive impact of QR codes on student motivation, autonomy and engagement in learning environments [3]. Studies indicate that students appreciate the convenience and efficiency of QR codes in accessing educational materials, contributing to a more interactive and practical learning experience [4, 5]. However, challenges regarding student familiarity with technology and the infrastructural requirements for effective implementation must be acknowledged, as they could hinder the adoption of QR codes in academic settings [6, 7]. Therefore, it is of interest to assess the impact of utilizing QR codes on the knowledge and perception of the participants and provide valuable insights into using QR codes.

## Methodology:

This mixed-method case-control study employed a rigorous mixed-method research design to evaluate the effectiveness and perceptions of Quick Response (QR) codes as teaching-learning tools used in the Department of Medicine for Phase 3 MBBS students at a medical college in Central India. All the students were divided into a control group, exposed to traditional manual access and an experimental group, exposed to Quick Response (QR) codes. The study was conducted at a medical college in Central India. As the participation was voluntary, a total of 115 Phase 3 MBBS students participated. 9 students refused to tender their consent for the study and were excluded. The remaining 106 students were divided equally into two groups, control and experimental.

## Inclusion criteria:

[1] Phase 3 MBBS students who had rotation duty in the medicine department.

[2] Willing to provide their written consent.

## Exclusion criteria:

Students who did not tender their written consent. Relevant studies material for online resources, lecture slides, videos, online quizzes and formative assessments and supplementary were identified. High-resolution QR codes were generated using Adobe Express's QR code generating tool (Adobe Inc., San Jose, California, United States). Pre-intervention and post-intervention knowledge evaluation and Feedback surveys were collected using Google Forms (Google Inc., California). These were reviewed by senior faculty members of the Medical Education Unit (MEU) and the Department of Medicine. Students' baseline familiarity with QR codes and their previous experiences with digital learning resources were captured using a Pre-intervention survey (10 questions designed). The post-intervention knowledge evaluation survey (25 multiple-choice questions (MCQs)) was administered one week after both control and experimental groups were exposed to the lab sessions. The feedback survey included 15 5-point Likert-scale questions examining students' perceptions of the usability, effectiveness and engagement of QR codes as teaching tools. Four focus group discussions were conducted with 8 to 10 students in each group, facilitated by a trained moderator. Each discussion was recorded and transcribed for analysis. Chi-square test was used to assess data distribution. A Paired T-test was used to assess the impact on knowledge between the control and intervention groups. A p-value less than 0.05 was considered significant. Thematic analysis was performed on transcripts of focus group discussions. A word cloud was generated to perform sentiment analysis on comments captured during the focus group discussion. Cronbach's Alpha was calculated to establish the consistency and reliability of the questionnaires used in the study. The Statistical analysis is performed using Python (v3.9) and its libraries, namely Pandas (v1.3.5), Seaborn (v0.11.2), WordCloud (v1.8.1) and SciPy (v1.7.1).

## Results/Observations:

Most of the study participants were in age groups between 20 and 24 years for both the control and experimental groups. 54.72% of the study participants were male. About 71.69% of the participants belonged to Tier 2 and Tier 3 cities. P-values of the group under all the categories are more than 0.05, i.e., not significant (Table 1 - see PDF). Most of the participants (83.02%) were using smart devices daily. Only one candidate rarely used it. About 84.90% of the students were trained in using computer/mobile applications or usage. 95.2% were familiar with some kind of online learning platform. 86.73% claimed to supplement their learning with medical learning through relevant apps. 98.11% of the participants were familiar with the QR codes, out of which the majority, 93.39%, used it for personal purposes. None of the participants had used the QR codes in medical education (Table 1 - see PDF). The average participant's knowledge of the control group was 2.67 and that of the experimental group was 3.38. The paired t-test's p-value, 0.004, clearly outlines significant improvement in the participants' knowledge (Table 2 - see PDF).

Feedback from the experimental group regarding the perception towards the usage of QR codes is presented in Table 3 (see PDF). Significantly large participants, close to 90%, either agreed or strongly agreed that QR codes enhanced my engagement with the learning materials, QR codes made accessing educational content more convenient, QR codes provided an interactive and dynamic learning experience, QR codes helped me connect theoretical knowledge to clinical practice, QR codes saved time in accessing and using study resources and The integration of QR codes in teaching was an innovative approach to learning. Though opinions were divided about the comfort of using QR codes as a part of the learning process, QR codes helped solve clinical problems more effectively and there was a preference for using QR codes over traditional methods for accessing learning materials. Transcripts of the focus group discussions reveal that many participants agreed that QR codes bring the curated material access at their fingertips and were largely positive toward using them as learning aids. A word cloud showcasing the sentiment analysis performed in comments captured from the focus group discussions is presented in Figure 1 (see PDF).

## Discussion:

This study aims to evaluate the effectiveness and perceptions of using QR codes as a teaching-learning tool by measuring the impact on knowledge and perception of the participants. Different studies have used QR codes for different purposes, ranging from medical education, clinical practice, patient care, public health & hospital administration, to research & documentation. Gender distribution (Male: Female) ratio in this study was 58:48. While a few studies had equal distribution, for the majority of studies it was predominantly tilted toward females [8]. The main reason for this as many such studies were carried out on nursing students [3]. The mean age of participants in most of the studies lay between 20-25 years [9]. The mean age of this study was 21-22 years. A significant increase in knowledge, from 2.67 to 3.39, was observed in this study. In a study performed by Bagheri-Nesami *et al.* [8] observed an increase in mean knowledge from 50.00 ± 22.82 to 59.66 ± 28.43. Al-Sababha [9] observed that the experimental group outperformed the control group in cognitive achievement and various skills. Datta *et al.* [10] noticed an increase in knowledge from 55.45 ± 10.04 to 60.03 ± 9.56. By analyzing the participant feedback, Minard [3] observed that participants found QR codes helpful in facilitating learning and maintaining attention during lab activities. Datta *et al.* [10] observed that students favor using QR codes to comprehend and evaluate museum exhibits. Bagheri-Nesami *et al.* [8] noticed significant improvement in the satisfaction level of the intervention group (75.80 ± 21.19) as compared to the control (68.00 ± 22.73). El-Sayad *et al.* [11] observed a significant rise in the overall perception score of the study group, from 47.37 ± 6.66 to 74.32±4.48, as compared to the control group, 47.66 ± 7.18 to 49.49±7.72. Mavropoulou [12] noticed that participants believed they were able to follow the lessons better and were motivated. As per the current study, participants strongly felt that QR codes offered an interactive and dynamic learning experience, content was relevant to their clinical learning, helped connect theoretical knowledge to clinical practice and saved time in accessing and using study resources. On the other hand, some students were hesitant in using QR codes and did not believe that QR codes help them solve clinical problems more effectively. Qualitative analysis performed by Bagheri-Nesami *et al.* [8] identified that factors affecting the level of satisfaction were user adoption of newer technology, exposure to QR codes and quality of mobile internet. Joshi *et al.* [6] noticed that despite various challenges, user satisfaction remains high, underscoring QR codes' effectiveness in optimizing healthcare delivery and outcomes from a human-centred perspective. As per a study by Diczbalis *et al.* [13], on patient education, QR codes were well received by both patients and anaesthesiologists. Marcus *et al.* [14] reported that QR codes increased student confidence and they found it engaging. AbuElEla [15] concluded that QR codes can improve students' engagement, satisfaction and perceived learning. The current study, using focus group discussions, revealed that QR codes enabled fingertip access to curated material and were considered positively as learning aids. This method was enthusiastically noticed for its novelty factors and the need to access them outside the labs can make it effective. On the other hand, doubts were raised on its role towards retention power. Mavropoulou [12] identified the shortcomings, such as problems with connectivity, battery, access, tuning and even scanning QR Codes. According to the current study, offline access and limited access to internet services or smart devices hinder the adoption of QR codes.

## Limitations and future scope of work:

The effect of using QR codes on the retention power of the participants was not evaluated. A long-term and multi-disciplinary study within the institute could bring forward more conclusive findings.

## Conclusion:

QR codes significantly enhance learning experiences, information retrieval and foster greater student engagement and knowledge is shown. Positive student feedback regarding usability and the convenience supports the broader adoption of QR codes in medical training. More dynamic, interactive and student-centred learning environment can be created by leveraging the benefits of QR codes and overcoming existing challenges. This in turn will better prepare future healthcare professionals.
